# Supplementary figures and images for: Comprehensive Metabolome and Transcriptome Analysis of Populus davidiana and Its Response to Drought Stress
Source: Biology (Basel). 2025 Nov 10;14(11):1574. doi: 10.3390/biology14111574 (PMC12649925; doi:10.3390/biology14111574)

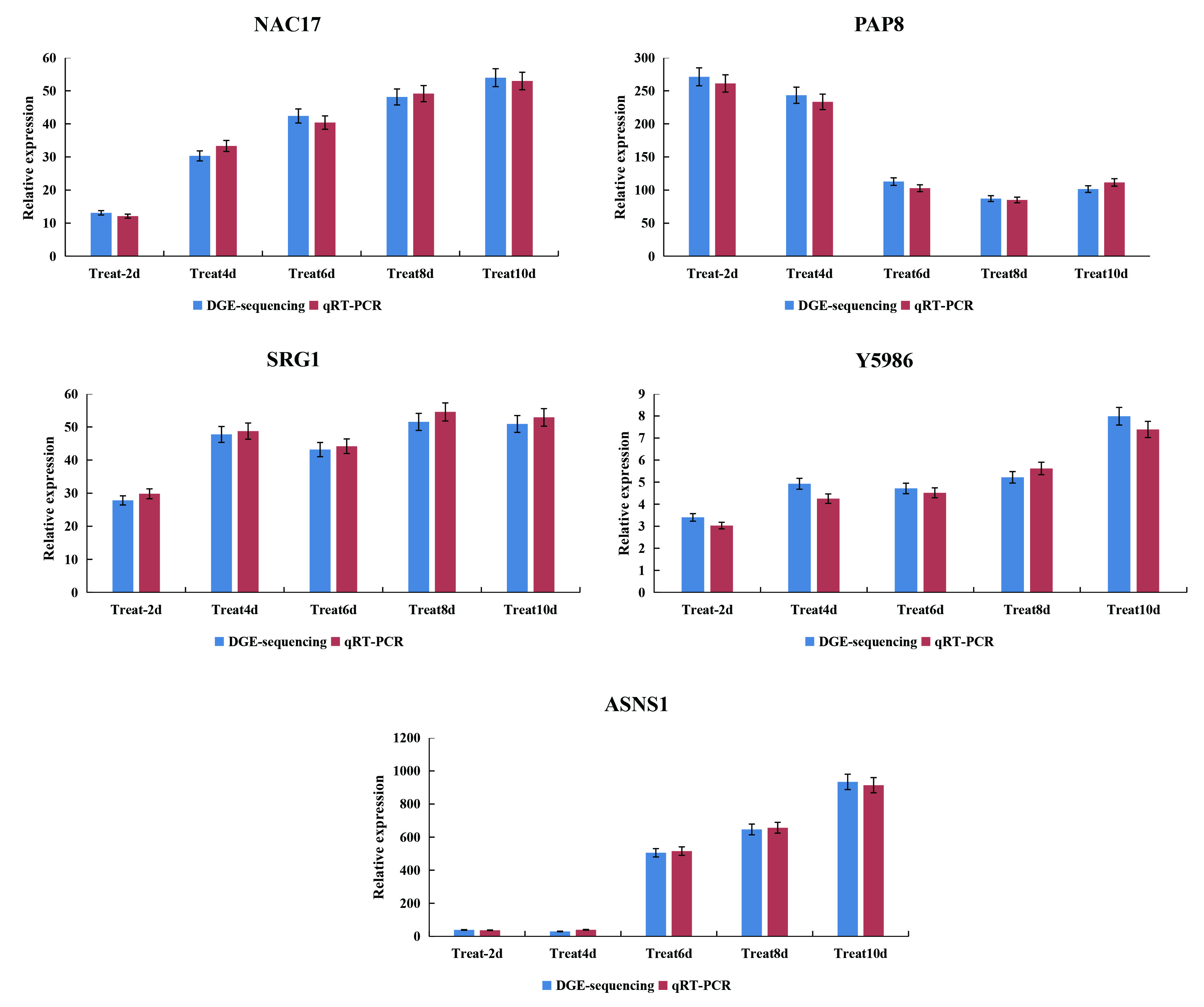

Supplement: Supplementary file 1 [file biology-14-01574-s001.zip › Figure. S1.jpg]
